# Practical advice in the development of a lyophilized protein drug product

**DOI:** 10.1093/abt/tbae030

**Published:** 2024-11-21

**Authors:** Yuan Cheng, Huu Thuy Trang Duong, Qingyan Hu, Mohammed Shameem, Xiaolin (Charlie) Tang

**Affiliations:** Formulation Development Group, Regeneron Pharmaceuticals Inc, 777 Old Saw Mill River Rd, Tarrytown, NY 10591, USA; Formulation Development Group, Regeneron Pharmaceuticals Inc, 777 Old Saw Mill River Rd, Tarrytown, NY 10591, USA; Formulation Development Group, Regeneron Pharmaceuticals Inc, 777 Old Saw Mill River Rd, Tarrytown, NY 10591, USA; Formulation Development Group, Regeneron Pharmaceuticals Inc, 777 Old Saw Mill River Rd, Tarrytown, NY 10591, USA; Formulation Development Group, Regeneron Pharmaceuticals Inc, 777 Old Saw Mill River Rd, Tarrytown, NY 10591, USA

**Keywords:** lyophilized protein drug product, early and late stage development, formulation optimization, primary container and closure selection, lyophilization cycle development

## Abstract

The development of lyophilized protein drug products is a critical and complex task in the pharmaceutical industry, requiring a comprehensive understanding of the myriad of factors affecting product quality, stability, and the efficiency and robustness of the lyophilization process. This review offers practical advice on the critical aspects of lyophilized protein drug product development. Practical considerations across both the early and late stages of development are discussed, underscoring the necessity of a strategic approach from initial development through to commercialization. The review then delves into formulation optimization strategies that are essential for enhancing protein stability and the efficiency of the lyophilization process. This section outlines stable formulation design and highlights the unique considerations required for high protein concentration lyophilized drug products. It further explores the formulation strategies to enhance the lyophilization process’ efficiency. Moreover, the paper examines the critical elements in selecting primary containers and closures for lyophilized drug products, focusing on vials and dual chamber systems. The analysis encompasses the effects of the container/closure’s material, size, geometry, and fill volume on product quality and process efficiency. Lastly, the review provides practical considerations in lyophilization cycle development, including the design and optimization of the freezing, primary drying, and secondary drying stages to achieve a robust, scalable, and efficient lyophilization process. By offering comprehensive insights into these key areas to enhance their understanding and implementation of best practices in the field, this paper serves as a useful resource for researchers, formulators, and process engineers involved in the development of lyophilized protein drug products.

## Teaser

Statement of Significance: This review offers practical advice on the critical aspects of lyophilized protein drug product development, including considerations across early and late stages of development, formulation strategies for enhancing protein stability and the efficiency of the lyophilization process, selection of primary containers and closures, and considerations in lyophilization cycle development.

## Introduction

Lyophilization, also known as freeze drying, is widely used for pharmaceuticals especially protein drug products to improve the stability and long-term storage shelf life of labile drugs [1, 2]. Lyophilized protein drug products have the advantages of good stability, long shelf-life, and easy handling for shipping and storage [3]. However, lyophilization is a time and energy intensive process that could take days or even weeks to complete if the product formulation and lyophilization process is not optimized [4, 5]. The characteristics of the lyophilized drug product and the stability of the drug during lyophilization process and storage [6] are the major considerations for the protein formulation and lyophilization process optimization.

For a stable product with acceptable quality characteristics, a short lyophilization cycle has the advantage of higher throughput with lower manufacturing cost. A sub-optimal drug formulation or lyophilization process may compromise drug stability, take longer and cost more than necessary to manufacture. In order to design an optimal drug formulation and lyophilization process, the formulation and process development scientists need to understand the critical properties of the formulation and how to apply this information to the formulation and process design. The critical formulation properties include the collapse temperature of the formulation, the stability of the protein drug, and the characteristics (e.g. cake appearance, moisture content and reconstitution time) of the lyophilized drug product impacted by the excipients used. The macroscopic collapse temperature of the formulation (T_c_) is the temperature above which the lyophilized drug product loses macroscopic structure and collapses during lyophilization [7]. T_c_ is usually a few degrees above the T_g_’, which is associated with the glass transition temperature in the frozen state or close to the eutectic temperature (T_eu_, the temperature at which a crystalline bulking agent mixture system melts) if solutes are crystallized in the frozen solution [8]. In a mixture of crystalline and amorphous freeze concentrate where the crystalline phase is in excess, the amorphous phase collapses if the product temperature is above T_g_’ (or collapse temperature of amorphous phase), but gross or “macroscopic” collapse will not occur unless the product temperature is above both T_g_’ and T_eu_. In order to produce an acceptable lyophilized drug product, it is always required to lyophilize a formulation at the temperature below the collapse temperature [1, 2]. The product quality attributes such as drug product stability, cake appearance, visible and subvisible particles, and reconstitution time are particularly important for protein formulations. Proteins are sensitive to the stresses imposed by lyophilization and are prone to degradation during the process and storage. The low temperature of lyophilization process does not guarantee protein stability because proteins could experience stresses of dehydration, cold denaturation or denaturation at interfaces (protein–air and protein–ice) [9–11].

Fortunately, thermodynamic instability caused by lyophilization stresses does not necessarily mean protein unfolding during lyophilization if the rate of unfolding is sufficiently slow on the time scale of the process in a viscous frozen formulation containing a sufficient amount of stabilizer(s) so that the process is complete before significant unfolding can occur. Therefore, the protein might safely be lyophilized at a temperature far above the T_g_’, allowing a much faster drying process. In general, to achieve an optimum formulation and lyophilization process with the highest drug quality and the least cost, it requires optimization of all aspects of drug formulation and all the controllable stages of lyophilization [12]. This review is intended to serve as a guide to rational formulation and process design and optimization.

## Practical considerations for early and late phase lyophilized protein drug product

In general, there are different considerations and preferences applicable to first in human or late phase lyophilized formulation (Table 1) and lyophilization process (Table 2). For an early phase lyophilized formulation, the major considerations are usually speed to clinic with limited material availability and product knowledge. A platform formulation and lyophilization process based on past product characteristics and formulation experiences can usually serve the purpose. The platform formulation and process approach can be relatively conservative to ensure successful technical transfer to different fill finish manufacturing sites, and fast development timeline with limited resource requirements although it may compromise process efficiency. When dealing with a novel modality where a platform formulation is unavailable, it is advisable to consider high-throughput screening-based formulation development, particularly when material availability is limited [13].

**Table 1:** Lyophilization formulation consideration for early and late phase lyophilized protein drug products.

**Factors to consider**	**Early Phase**	**Late Phase/Commercial**
Material and Time Limitations	Limited material and compressed timeline	Comprehensive formulation development and optimization with less limit on material and time
Product Knowledge	Limited	Good understanding and development experiences
Formulation	Preliminary and/or platform	Optimized formulation for drug stability and meets the requirement for intended administration route. (such as, isotonic, short reconstitution time, low viscosity, elegant cake appearance)
Excipients (that improve cycle efficiency)	Usually not needed for speed to clinical	Important for lyophilization formulation with extended cycle time (e.g. > 3 days)
Stability	2°–8°C storage for clinical supply	≥ 24 month 2°–8°C storage + ≥ 30 days room temperature TOR for commercial distribution, storage stability and shelf life Room temperature stable (ideally)

**Table 2:** Lyophilization process consideration for early and late phase lyophilized protein drug products.

**Factors to consider**	**Early Phase**	**Late Phase/Commercial**
Material and Time	Limited material and tight timeline	Allow more comprehensive development
Lyophilization Site and Capacity	May change between different CMOs and/or different lyophilization units at the same manufacturing site	Selected/known manufacture site and lyophilization unit
Product Knowledge	Limited process development	Good process understanding with comprehensive development experiences
Economic/cost	Less important (only a few runs to support early clinical trials)	Important (repeat runs for larger scale clinical trials and commercial supplies)
Lyophilization cycle	Conservative/Platform and can fit different lyophilization units	Optimized/Efficient/Robust with defined design space for selected lyophilizer unit(s) intended for commercial production

In contrast, the late phase formulation and lyophilization process are designed and optimized for product quality and process efficiency at commercial scale with a full consideration of formulation and process robustness and product cost effectiveness. A full characterization of the lyophilization process with an acceptable design space is often desired for a commercial lyophilized drug product, which can be achieved through lyophilization process quality by design (QbD) and design of experiment approaches [14]. Considerable material, time, and resources are required to optimize and characterize the formulation and lyophilization process. For example, the late phase lyophilized drug product usually requires 24 months or longer shelf-life at 2°–8°C with a 30-day room temperature time out of refrigeration (TOR) for commercial distribution/storage, acceptable room temperature post reconstitution stability for administration or maybe even stable for room temperature storage and distributions.

The formulations of the late phase and commercial products are often optimized to have a relatively high collapse temperature, which makes it more efficient and easy to lyophilize. The osmolality of the reconstituted drug product should be close to isotonic with acceptable viscosity for the intended route of administration (e.g. subcutaneous injection) and the reconstitution time is relatively short for clinical convenience.

## Formulation optimizations for protein stability and lyophilization process

### Lyophilization process associated stresses

During the lyophilization process, proteins are subjected to multiple stresses that can adversely affect their stability. These include cold denaturation, freeze-concentration, ice-water interface, phase separation, and dehydration. This section will explore the primary stresses encountered during lyophilization.

#### Cold denaturation

Cold denaturation refers to the spontaneous protein unfolding at the temperatures typically well below zero degrees Celsius [15]. Cold denaturation is largely an enthalpy-driven process, arising from the increased solubility of non-polar groups and the consequent weakening of the hydrophobic core of proteins at lower temperatures [16]. In contrast, the thermal denaturation is mainly an entropy-driven process [17]. Cold denaturation can occur during the freezing process of lyophilization, where the product temperature is often lowered to −20°C to −50°C. Proteins are more resistant to cold denaturation at higher protein concentrations or in the presence of stabilizers such as sugars and polyols [9]. The rate of cold denaturation is typically slow on the timescale of freeze-drying, allowing primary drying to be conducted above T_g_’ [18].

#### Freeze-concentration

Freeze-concentration refers to the phenomenon where the solute concentration dramatically increases during freezing [5, 19]. A dramatic increase in salt concentration can reduce protein colloidal stability and lead to protein aggregation [20]. A significant increase in protein concentration can increase the tendency of protein aggregation [21, 22]. Freeze-concentration can also increase the concentration of impurities in the formulation such as metals, reactive oxygen species and proteases, accelerating protein degradation [23]. Differential crystallization of buffer salts resulting from freeze concentration can lead to dramatic shift in pH (≥ 3 pH unit) [24, 25]. pH can also change due to temperature dependent pKa values for amine-based buffer systems, such as histidine, TRIS and etc. Additionally, excipients may crystalize or precipitate when frozen.

#### Phase separation

The formulation components, such as buffer salts, stabilizers and bulking agents, can separate into distinct phases during lyophilization. Liquid–liquid phase separation has been reported for many systems relevant to protein formulation, such as polymer-polymer, polymer-salt and protein- polysaccharides [26, 27]. Liquid-liquid phase separation can lead to the depletion of stabilizers from proteins and therefore compromise protein stability [25, 28]. Crystalline-amorphous phase separation can also occur when buffer salts, bulking agents and stabilizers crystallize due to freeze-concentration [29]. Crystallization of bulking agents such as mannitol and glycine from amorphous proteins can lead to the loss of their stabilizing effect [30].

#### Ice-water interface

Proteins are exposed to ice-water interfaces during the freezing process of lyophilization. The area of ice-water interfaces depends on the number, size and morphology of the ice crystals. Direct adsorption of proteins to the ice-water interface can lead to protein denaturation and aggregation [10, 31]. In addition to direct adsorption, the ice-water interface can promote the cold denaturation of proteins by weakening the hydrophobic core that is critical for maintaining protein structure [32].

#### Dehydration

Proteins experience dehydration stress during the drying process of lyophilization when water molecules bound to the protein surface are removed [33]. Water molecules bind to protein surface via hydrogen bonding, and this interaction plays an essential role in stabilizing protein structure [33, 34]. Disruption of water-protein interactions can lead to protein denaturation and aggregation [35]. Stabilizers such as sugars and polyols that can replace water molecules to form hydrogen bonds with amino acid residues at protein surfaces, thereby protecting proteins against dehydration stress [35, 36]. In contrast, polymeric stabilizers like PEG are not effective against dehydration stress due to their inability to replace the hydrogen bonds between protein and water [37].

### Considerations for the development of stable lyophilized protein formulations

#### Buffers

The selection of buffers is critical for the development of lyophilized protein formulations. A pH screening is typically conducted to identify the optimal pH when developing a lyophilized protein formulation. It is important to note that the stability of the liquid form of a lyophilized protein formulation should be considered when determining the optimal pH. It is preferable to select buffers, such as histidine, Tris and citrate, that do not crystallize during freezing Volatile buffers, such as acetate, are not suitable for lyophilized formulations. To avoid dramatic pH change resulting from differential crystallization of buffer components [38], it is recommended to avoid the use of high concentration phosphate salt buffers such as sodium phosphate. When the use of phosphate salt buffers is necessary, the risk of buffer crystallization can be mitigated by minimizing the buffer concentration (≤ 20 mM) or adding excipients that can inhibit the crystallization of phosphate salts, such as trehalose and sucrose.

#### Stabilizers

To maintain protein stability during lyophilization and long-term storage, stabilizers are typically an essential component of lyophilized protein formulations. The most effective stabilizers for lyophilized protein formulations are sugars, such as sucrose and trehalose. Other stabilizers for lyophilized protein formulations include amino acids or sugar alcohols, such as mannitol and sorbitol, and cyclodextrin. Sugars can protect proteins against various stresses during lyophilization (e.g. cold denaturation, ice-water interface, freeze-concentration and dehydration) and long-term storage. Trehalose can crystalize from an aqueous solution during freezing. The β, β-trehalose crystallizes much more rapidly from a freezing aqueous solution than does the α-α trehalose [39].

The stabilizing effect of sugars in liquid state (or freeze-concentrates) is generally explained by the preferential exclusion mechanism [40, 41]. The preferential exclusion of sugars from protein surface makes it thermodynamically favorable for proteins to exist in folded state since protein unfolding will increase the total surface area of proteins. The stabilizing effect of sugars in solid-state can be attributed to the formation of sugar glass and the replacement of hydrogen bonding between protein and water [42]. The formation of sugar glass greatly reduced the mobility of molecules and the rate of physical and chemical degradation. Sugars can act as water substitutes and form hydrogen bonds with proteins in the place of water, which is essential for maintaining the structure and conformation of protein [42].

Increased protein stability is typically observed at a higher stabilizer to protein molar ratio for lyophilized products. It is worth noting that reducing sugars, such as glucose and lactose, should be avoided in the selection of stabilizers. While reducing sugars may be effective at preventing protein folding, they tend to react with proteins via Maillard reaction, leading to protein glycation that can adversely affect product quality [6].

Polymeric stabilizers such as PEG and dextran can be used to increase the collapse temperature and therefore achieve better cake structure or shortening drying time. Polymeric stabilizers are generally effective at preventing protein unfolding during freezing; however, their stabilizing effect can be negated if phase separation occurs during the freezing process [43]. As previously mentioned, phase separation has been observed in numerous formulations containing polymers [26, 27]. Polymeric stabilizers often fail to stabilize proteins during the drying process, presumably due to the inability to substitute water in forming hydrogen bonds with proteins. Studies have shown that the combination of polymeric stabilizers and disaccharides can provide good stabilizing effect [6].

#### Surfactants

Surfactants are often included in lyophilized protein formulation to protect proteins against interfacial stresses from ice-water interfaces during freezing and air-liquid interfaces during reconstitution. It is generally recommended to maintain the surfactant concentration above its critical micelle concentration to achieve sufficient stabilizing effects [44]. Meanwhile, it is advisable to limit the concentration of surfactants to mitigate the risk of protein degradation linked to surfactants or their impurities. Peroxides, which can be introduced into protein formulation as residual impurities from polysorbate or as by-products of polysorbate auto-degradation, can cause protein oxidation and aggregation during lyophilization [45–49].

#### Viscosity reducers

Viscosity reducers such as arginine hydrochloride, sodium chloride and proline may be considered when the viscosity of the formulation is a concern. Arginine hydrochloride is a commonly used viscosity reducer for lyophilized formulation due to its effectiveness and its tendency to remain in a single amorphous phase with proteins during lyophilization [50]. In contrast, sodium chloride has a much higher tendency to crystallize, leading to its separation from the amorphous phase containing proteins [51].

#### Bulking agents

Crystalline bulking agents such as mannitol and glycine should be considered when there is a challenge in forming mechanistically strong and elegant cake [6]. These bulking agents can crystalize and provide support to cake structure. While crystalline bulking agents typically do not protect protein against lyophilization stress, it has been demonstrated that the mixing of mannitol and glycine at certain ratios can lead to partial crystallization. The fraction of mannitol and glycine remaining in amorphous phase can provide sufficient stabilizing effect [6]. Crystalline mannitol hydrate formed during lyophilization can release water to the amorphous protein drug during storage, potentially compromising the stability of the lyophilized protein formulation.

#### High protein concentration formulations

The development of high protein concentration lyophilized formulations often presents unique challenges, such as high opalescence in the reconstituted drug product, long reconstitution time and the need to balance between stability and osmolality. Special considerations in formulation design are required to address these challenges effectively. Tang et al has discussed in detail the challenges and considerations in the development of high protein concentration lyophilized formulations [52].

### Formulation strategy to improve lyophilization efficiency

Primary drying is generally the most resource-intensive process, accounting for ~ 40% of the total resource required for a lyophilization process [53]. A higher shelf temperature can accelerate the rate of sublimation and shorten the time required for primary drying. For example, a 1°C increase in product temperature during primary drying can lead to a 13% reduction in primary drying time [1]. To produce lyophilized protein products with satisfactory appearance and long-term stability, the product temperature typically needs to be maintained at 2–3°C below the collapse temperature. Therefore, the formulation should be optimized to achieve the highest collapse temperature to shorten primary drying.

One strategy to increase collapse temperature is to increase protein concentration in the formulation. Depaz *et al.* reported that the collapse temperature of two monoclonal antibodies increased from approximately −30°C to approximately −20°C when the protein concentration was increased from 0 to 100 mg/ml [54]. However, an increase in protein concentration may require higher stabilizer concentration, which can result in a significant increase in total solid content. This can lead to difficulty in reconstitution and primary drying.

Another strategy to increase the collapse temperature is to add crystalline bulking agents, such as mannitol and glycine. A drawback of this strategy is that a higher bulking agent to stabilizer ratio is required to ensure their crystallization prior to the primary drying. Since the crystalline bulking agents are typically used as fillers to achieve desirable cake structure and do not provide significant stabilizing effect, a low bulking agent concentration is preferred. It has been reported that crystallizing amino acids such as phenylalanine, leucine and isoleucine can serve as alternative bulking agents. These amino acids can crystallize at a lower bulking agent to stabilizer ratio, enabling fast drying at low bulking agent concentrations [55].

## Container closure consideration

Selection of primary containers and closures is a critical aspect for lyophilized drug product development. Primary containers for lyophilized drug products include vials/bottles, ampules, dual-chamber syringes and cartridges. Historically, ampules have been used for lyophilized drug products due to their superior sealing. It has been much less frequently used now due to the potential of introducing glass particles into drug products when breaking the ampule seal. Type 1 glass vials remain the most common primary containers for lyophilized drug products. General requirement for glass containers used in pharmaceutical products are elaborated in USP < 660> and Ph. Eur. Chapter 3.2.1. Recently, dual-chamber systems offer the convenience of direct reconstitution. The discussions in this section primarily focus on vials and dual-chamber systems.

### Vials and closures

The major factors in selecting vials and closures are summarized in Table 3. The typical lyophilization steps, including freezing, primary drying, and secondary drying, require adequate heat transfer to and from both the formulation and primary container/closure. During lyophilization, flow of water molecule is impeded by resistance from the dried-product layer, semi-stoppered vial, and sometimes the duct between chamber and condenser [56]. A heat transfer coefficient, *K*_v_, is commonly used to describe the relationship between heat flow and temperature difference between the heat source (the shelf) and the heat sink (the product) during primary drying. For a typical vial product lyophilization, the heat is transferred through three main pathways: (i) the heat conduction from the shelf to the vial through direct contact (*K*_c_), (ii) the heat convection through the air mainly between the vial bottom and shelf (*K*_g_) which is usually the primary heat transfer during ice sublimation, and (iii) radiative heat transfer (*K*_r_). The primary heat transfer contribution of mechanism is dependent on both chamber pressure and the type of primary container. Therefore, the *K*_v_ (Equation 1) derived from these three heat transfer mechanisms (*K*_c_ + *K*_g_ + *K*_r_) is an important parameter in primary container selection [56].


(1) \begin{equation*} {K}_{\mathrm{v}}={K}_{\mathrm{c}}+{K}_{\mathrm{g}}+{K}_{\mathrm{r}} \end{equation*}

**Table 3:** Factors for selecting vials and closures for lyophilized drug product.

**Component**	**Factor**
Vial	Material compatibility with formulation Heat transfer properties (material, bottom geometry, wall thickness, size) Extractable and leachable profile Susceptibility to breakage Machinability Oxygen permeability Fill volume Color/light protection Other functions (such as inner coating to improve cake appearance)
Closure	Material compatibility with formulation, surface interaction Moisture permeability/absorption Water vapor flow (venting design) Extractable and leachable profile Fitting with vial neck design to provide good sealing Machinability and surface stickiness Coring potential (e.g. needle, spike) Resealable property Balanced hardness permitting stopper insertion and needle penetration

#### Vial material

Glass vials are commonly used as the primary container for lyophilized biopharmaceutical drug products. Type I glass is generally compatible with proteins, peptides, oligonucleotides, as well as small molecule formulations. It also provides good moisture and oxygen barrier and has superior heat transfer properties suitable for lyophilization. Amber glass vials are available for light-sensitive products. Glass vials specifically designed for lyophilized products may contain a hydrophobic inner surface coating, comprised of non-covalent silicone oil or a covalent hydrophobic silicone to improve cake appearance, reduce fogging, and maximize withdrawable volume [57].

Polymer vials, commonly made of COP (cyclic olefin polymer), COC (cyclic olefin copolymer), and polypropylene have also been used as the primary containers. Polymer vials offer the advantage of resistance to vial breakage with minimal leaching. However, polymers are generally less efficient in conducting heat (with lower *K*_v_ value than glass vials) [58], which potentially requires a longer primary drying time. Oxygen and water vapor could permeate through conventional polymer vials. Novel technologies, such as adding polyamide oxygen barrier layer (COP/polyamide/COP) improved barrier properties. Other inner and outer silica-based hydrophobic coatings could also act as a gas barrier to minimize oxygen or water vapor exposure [59].

Hybrid vials using polymer material with glass (silicon dioxide) coating inside could also be used as the primary container for lyophilized vials. These vials are molded with a flat bottom to improve heat transfer. The lyophilization processing time using this type of hybrid vials is comparable to borosilicate vials with minor adjustments to the process parameters [59].

#### Vial size and fill volume

Standard vial sizes for lyophilized drug product range from 2 mL to 100 mL. The selection of vial size for DP depends on fill volume and post-reconstitution volume, which are determined from the target dose and the formulation concentration. Typically, the fill volume in the glass vial is no more than 35–40% of the vial capacity to avoid vial breakage. Hybrid COP vials potentially could be filled to higher fill volumes without the risk of breakage [58].

#### Vial geometry

Studies have shown that the gap between the vial bottom and shelf contributes significantly to heat resistance and vial bottom geometry has a great impact on heat transfer [60].

There are two types of glass vials based on manufactured process, tubular vials and molded vials. Tubular glass vials are converted from glass tubing where the wall thickness is controlled and the vial base shape can be optimized during conversion of the tubing to base. Molded glass vials are formed by pouring liquid glass into a mold. Traditionally the molded glass vials often have a thicker wall in contrast to tubular vial and a concave bottom. With wide temperature ranges during lyophilization, a uniform thickness of the glass vial bottom and side with a low coefficient of expansion is desirable. The concave bottom of the vial prevents the close contact and leaves an air-filled gap between the vial and the freeze-dryer shelf. The air gap may limit the heat transfer during lyophilization, lowering *K*_g_ (Equation 1) and resulting in inefficient heat transfer. However, in the last decade, there have been improvements in molded glass vial manufacturing, such that the bottom geometry of the molded vials could be comparable to tubing glass vials, resulting in improved heat transfer performance and similar *K*_v_ [61–63].

There are three types of neck design for glass vials, American blowback (ABB), European (blowback (EBB), and no blowback (NBB). The ABB and EBB vials reduce the risk of stopper pop-off, which are important for lyophilization application.

#### Closures

The vial closure (stopper) for lyophilized drug products serves two functions: venting sublimated ice and water vapor with partial insertion during lyophilization and closing the vial after lyophilization. The factors for selecting closures are outlined in Table 3. For lyophilized drug product, which is often sensitive to moistures, selecting stoppers containing low moisture and with low moisture vapor transmission (MVT) is important to ensure product quality and stability. Stoppers may absorb trace amount of moisture from stopper manufacturing, sterilization, storage, and even during drug product lyophilization. The trace amount of moistures contained in the rubber stoppers may be transferred to the lyophilized product [64].

Stoppers made of butyl or halogenated butyl (bromobutyl or chlorobutyl) elastomer are commonly used for vialed lyophilized drug products. These elastomers are low in gas permeation, low in moisture absorption and MVT, and provide low coring, good sealing and resealing properties. When used for lyophilized biopharmaceutical drug products, the stoppers are usually coated with fluoropolymer (such as tetrafluoroethylene and ethylene coating, or polyethylene and tetrafluoroethylene coating) to improve drug compatibility. The coating minimizes the extractable and leachable from the rubber components, reduces moisture transmission, and also modifies the surface properties of the stoppers [65]. The coating on the side and top of stoppers, if presents, prevents stickiness and avoids the need for siliconization.

The configuration of the stoppers is especially important for lyophilization. Typically, a stopper for lyophilized product has a vent opening for sublimated ice, and it has 3 types of design, single vented (igloo), dual vented, or three-legged configurations. The single-vented stoppers are commonly used attributing to ease of handling and good machinability. Vial stopper plugs also come with many different designs, such as different height, diameter, vent position, rib design, blowback, and rings. The stopper design should match the vial neck to ensure proper sealing [66].

### Dual-chamber systems

There are several lyophilized drug products with dual-chamber systems on the market, including vials, syringes, and cartridges. The use of dual-chamber systems improves the convenience of use as well as patient safety, but the manufacturing process could be complex [67].

**Figure 1:**
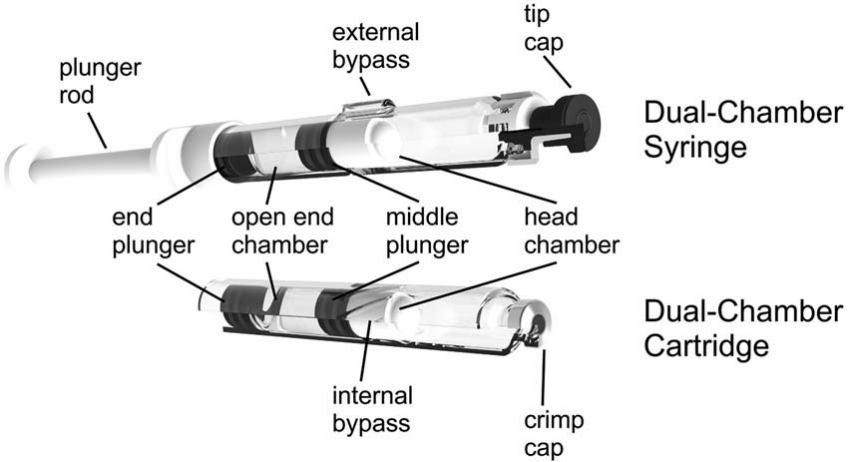
Schematic diagram of dual-chamber device. Adapted from [68]. The lyophilized cake is stored in the head chamber and the diluent is stored in the open end chamber.

Dual-chamber vial, also called act-o-vial, is a vial with two compartments, lyophilized drug product compartment and diluent compartment separated by a stopper. Dual chamber syringes and cartridges, consisting of a barrel which is divided into two chambers via a plunger, with one chamber containing the lyophilized product and the other chamber containing the diluent, are becoming more common (Fig. 1). The dual chamber cartridge is similar to syringe, except that the cartridge is sealed with a septum and the syringe is closed with a Luer cone and needle. The diluent can bypass the stopper upon activation via an external channel or internal groove for reconstitution. Dual chamber syringes and cartridges offer accurate dosing with minimized steps for reconstitution.

Both glass and polymers (COP or COC) have been used as primary container for dual chamber cartridge or syringe barrels. Glass barrels are compatible with most drug formulations and manufacturing process but are prone to breakage. Polymer barrels offer the advantage of resistance to breakage and safety especially when dual chamber systems involve using devices (such as autoinjector), and therefore gained more acceptability recently.

One of the key components in the dual-chamber system is the middle plunger, which separates the barrel and is always in contact with the formulation and diluent during storage. The plunger must have a low moisture sorption potential during storage and low leachable profile to ensure the quality of the lyophilized drug. The plungers are usually coated with fluoropolymer coating to minimize leaching. 1 ml dual chamber syringe or cartridge is the most common size for commercial products on the market. The dual chamber devices are also available in 1–5 ml and potentially > 5 ml. The selection of the dual chamber devices is dependent on the dose volume. The lyophilization of dual chamber syringes and cartridges are different from vials. The syringes and cartridges are usually not in direct contact with the freeze-dryer shelf and the heat transfer is primarily by convection rather than conduction. A few review articles have summarized the lyophilization for dual chamber systems [69, 70].

## Lyophilization cycle development for protein drug product

Developing a lyophilization cycle for a protein drug product involves a series of meticulously planned steps to ensure the product’s stability, efficacy, and quality. Besides formulation optimization, the parameters of the lyophilization cycle play key roles and must be carefully optimized to achieve long-term stability, a reasonable reconstitution time, and process efficiency. Therefore, it is crucial to build a controllable cycle that can be revised and improved for different scales of lyophilization. Optimization of the cycle should encompass a comprehensive evaluation of all three stages inherent to the lyophilization process [12].

### Freezing stage

Freezing is the first step of the lyophilization cycle, solidifying the protein formulation by turning it from liquid to solid at a temperature below glass transition temperature T_g_’ [1, 71]. Controlled nucleation is a freezing technique that can manage super-cooling to control ice crystal size and distribution. By inducing ice formation at a specific temperature, it ensures uniform ice crystals across all vials, resulting in a consistent and homogeneous product. There are several methods to achieve controlled nucleation, including a) ice fog method that involves introducing a fog of small ice particles into the product, acting as nucleation sites and inducing the formation of ice crystals; b) pressure drop method that involves pressurizing and reducing the pressure suddenly in the lyophilizer chamber, causing ice formation at the controlled ice nucleation temperature; and c) electromagnetic field method that involves using an electromagnetic field to induce nucleation. By controlling the nucleation process, manufacturers can optimize lyophilization cycle time, product consistency, and transition from the early phase to the late phase. Among all the above controlled nucleation methods, the pressure drop method has been successfully adopted for GMP productions of lyophilized drug products [70, 71]. Annealing is an optional step that holds the protein formulation at a specific temperature above the T_g_’ for a defined period after the freezing step. This process allows for the growth and reorganization of ice crystals and leads to larger and more uniform ice crystals, which can enhance the sublimation rate and reduce the drying time. This step is particularly beneficial for formulations that require the crystallization of solutes, such as mannitol or glycine, to avoid amorphous phases and enhance product stability [12]. Therefore, the freezing process needs to be carefully controlled and optimized to ensure the quality of the final product and the efficiency of the lyophilization process.

### Primary drying stage

The principle behind primary drying is ice sublimation. In this stage, the lyophilizer chamber is placed under a vacuum that is below the vapor pressure of ice, heat is then applied to sublimate the ice. This process removes about 80–95% of the water in the protein product while maintaining its physical structure. The fundamental concept of primary drying is to select the optimal target product temperature (T_p_), a few degrees below the collapse temperature T_c,_ to ensure the cake structure and efficient sublimation [7]. Shelf temperature plays a crucial role in the primary drying and it is typically set above the target T_p_ to provide adequate heat for ice sublimation. The chamber pressure (P_c_) controls the rate of sublimation and influences both heat and mass transfer. Consistently maintaining a chamber pressure below 50 mTorr is challenging, and there is little benefit in exceeding 200 mTorr. It has been reported that moderate chamber pressure (100–150 mTorr) provides optimal homogeneity in heat transfer among vials. Therefore, the ideal chamber pressure balances a high sublimation rate with uniform heat transfer. Equation (1) can be used to determine the “optimal” chamber pressure for a given T_p_ [12].


(1) \begin{equation*} {\mathrm{P}}_{\mathrm{c}}=0.29\times{10}^{\left(0.019\times \mathrm{Tp}\right)} \end{equation*}

The recommended relationship between the target T_p_, the vapor pressure of ice pressure at sublimation interference (P_ice_), and the recommended chamber pressure (P_c_) to achieve optimal sublimation are summarized in Table 4. Ramp rate is another parameter of the primary drying stage that refers to the rate at which the temperature is increased or decreased on the shelves that hold the product. Usually, a low to moderate ramping rate (e.g. 0.1–1°C/min) is implemented during the freezing and primary drying stage considering the cooling/heating capacity of the lyophilizer and the process efficiency. The goal is to ensure that the temperature increase does not exceed the cake’s ability to maintain its structural integrity [12, 14]. In general, the pressure within the lyophilizer chamber is monitored via a capacitance manometer and is controlled by the introduction of nitrogen during the primary drying phase. When the gas composition in the lyophilizer chamber changes from mostly water vapor to mostly nitrogen, it shows that primary drying is almost completed. A method for endpoint determination involves measuring the setpoint pressure and a Pirani gauge that measures the gas’s vacuum pressure-dependent thermal conductivity. Throughout the primary drying step, the Pirani gauge displays a higher value than the acute chamber pressure, indicating ice sublimation within the chamber. At the end of the primary drying, the pressure measured by the Pirani gauge decreases and aligns with the pressure recorded by the capacitance manometer [72]. The chamber pressure data are collected during the test and can be used to evaluate the endpoint detection by comparing with established criteria, which are dependent on the formulation components, drying condition, and previous experience [72–74]. The ice sublimation needs to be completed before starting the secondary drying to ensure no cake meltback occurs. An incomplete primary drying step can lead to several issues, such as decreased protein drug product stability, high levels of residual moisture that can cause chemical degradation through hydrolysis, collapse of the cake structure, and the possibility of batch failures. Additionally, there might be significant unfrozen water after the primary drying stage. A sample thief can be used to withdraw samples and monitor drying progress and quality without interrupting the process [50].

**Table 4:** The relationship between the target product temperature (T_p_), the vapor pressure of ice pressure at sublimation interference (P_ice_), and the selected chamber pressure (P_c_).

T_p_ (°C)	P_ice_ (mTorr)	Selected P_c_ (mTorr)
−10	2000	200
−15	1200	150
−20	770	120
−25	470	100
−30	290	80
−35	170	60
−40	96	50

### Secondary drying stage

This is the stage after primary drying. In this process, residual moisture remaining in the product is removed by desorption. This step uses a higher temperature, typically near or slightly above room temperature but below the glass transition temperature of the product (T_g_) with a relatively slow ramping rate [74]. Additionally, the chamber pressure is maintained at the same level as that used during the primary drying phase. Generally, temperatures and pressures in the range of 20°C to 40°C and 60 to 150 mTorr, respectively, are common, but they can be adjusted based on the needs of the drying process and product properties. Normally, drying time for secondary can vary from 3 to 12 h and it varies greatly depending on several factors, including the type of product, its moisture content, the temperature, and the pressure conditions. Just like in the primary drying step, a sample thief is useful for assessing the relationship between shelf temperature, time, and residual moisture, aiding in quality control and process optimization by providing data to refine future lyophilization parameters [50].

**Figure 2:**
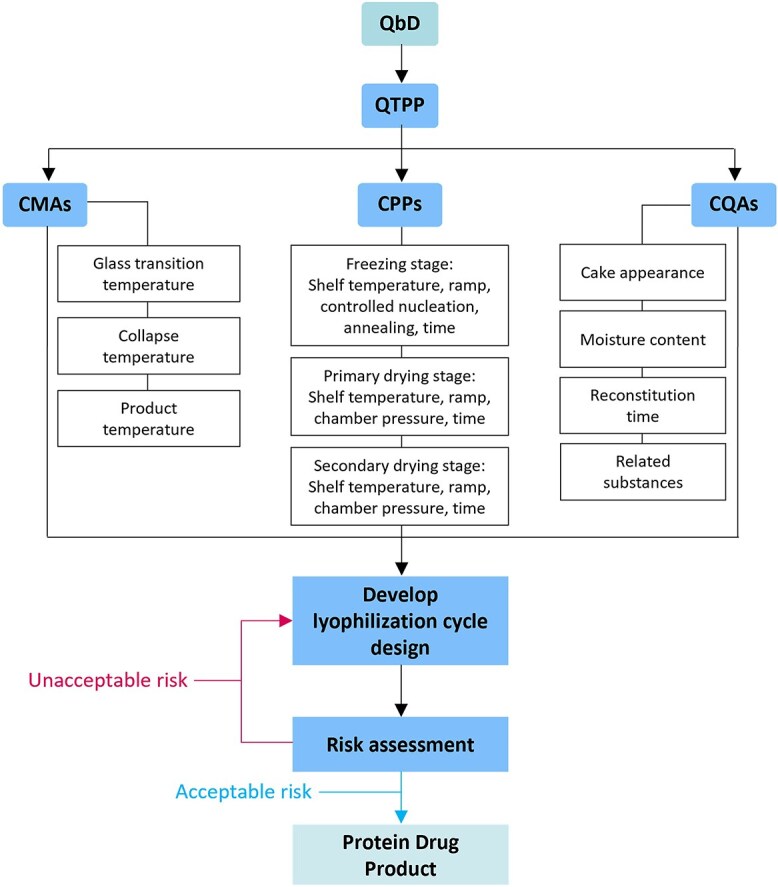
Overview of QbD approach for lyophilization cycle development. Adapted from [76].

### Lyophilization strategy to improve protein shelf-life and storage stability

The development of a controllable and robust lyophilization process is crucial to improve the shelf life and storage stability of protein drug products. It is common to study the stability of reconstituted liquids at 2–8°C or room temperature for short periods to ensure efficacy and safety. This article provides valuable insights for healthcare professionals and ensures the product meets quality standards throughout its shelf life. There is a systematic approach that focuses on understanding and controlling processes to ensure they meet the specific critical quality attributes (CQAs) of a protein drug product. This approach is referred to as QbD which leverages knowledge and risk management principles to develop a robust lyophilization process. It ends with a targeted plan that focuses on the essential steps and assessments needed to achieve the product’s quality [75]. CQAs, critical process parameters, and critical material attributes are the fundamental components of a QbD approach (Fig. 2) [77].

**Figure 3:**
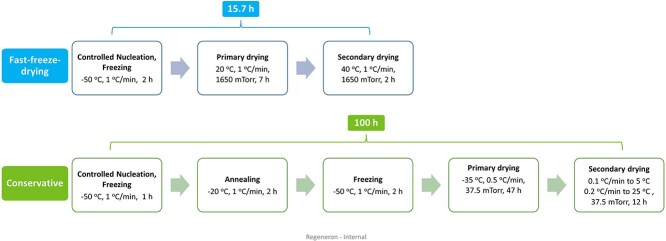
Lyophilization conditions variations of protein formulations. Adapted from [78].

Arsiccio and colleagues proposed a design space approach that may be used in the QbD process to guide the selection of optimal freezing conditions, with a focus on protein stability [77]. They found that the main cause of protein denaturation is the interface between ice and water, and the length of the freezing process significantly impacts protein stability. Wolfgang’s team recently focused on creating a fast-freeze-drying cycle for protein formulations containing mannitol and sucrose. The findings of this study indicate that fast-freeze-drying not only achieved protein stability throughout the process but also yielded a desirable cake appearance, significantly reducing the time required, in comparison to a conservative cycle (Fig. 3).

**Figure 4:**
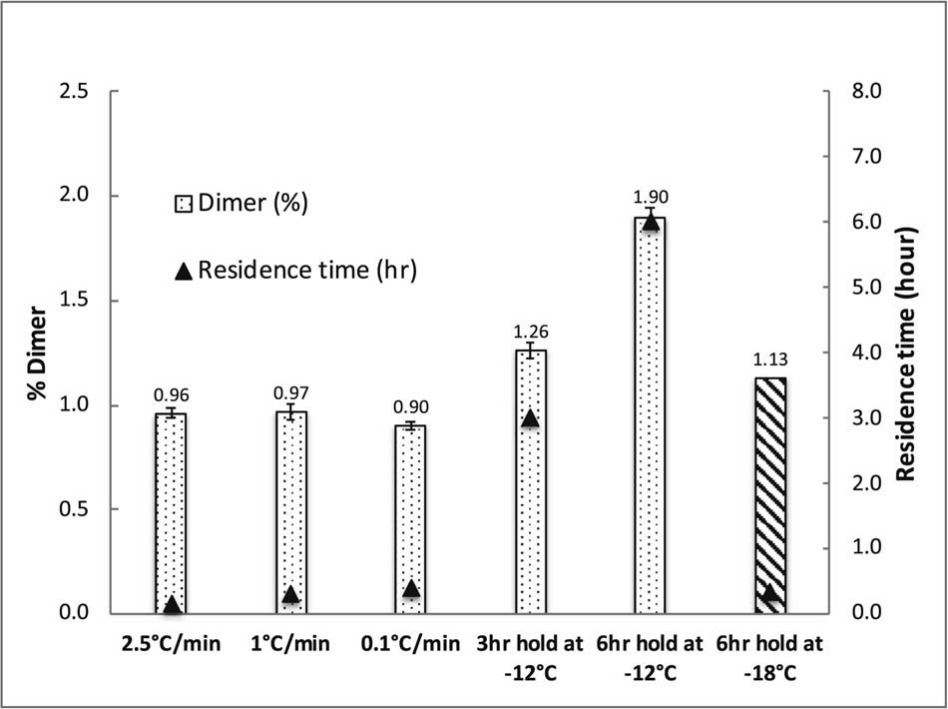
%soluble aggregation of rHSA (3 mg/ml) measured by SEC as a function of residence time (indicated on the right axis) at various process conditions. Only dimers were detected by SEC in the reconstituted rHSA samples after freeze-drying at all cooling rates. In all experiments, ice nucleation was controlled at a shelf temperature of −12°C. The shelf ramp rate post ice nucleation varied from 2.5°C/min to 1, or 0.1°C/min. For the studies of hold time and hold temperature, % soluble aggregation was measured for a 6-h hold post-ice nucleation at −12°C or −18°C. The residence time in the frozen concentrate was calculated from when the ice nucleated to when the product temperature reached −12°C. Reproduced from [79].

Rui Fang and their team investigated how initiating ice formation at higher temperatures reduces protein aggregation at the ice/water boundary [79]. By controlling ice formation at a higher shelf temperature (specifically, −5°C), they observed the formation of larger pores and channels in samples of recombinant human serum albumin (rHSA) and human immunoglobulin G (IgG) with 10% sucrose, indicating a decrease in ice surface area which could potentially lower protein aggregation. In addition, the study highlighted the significance of the time proteins spend in the freeze-concentrated phase during the freezing stage as a critical factor influencing protein stability (Fig. 4).

Enhancing the shelf-life and storage stability of lyophilized protein drug products via lyophilization involves a holistic strategy that includes careful process optimization. Key factors such as controlled freezing and precise timing of the lyophilization cycle are crucial. These elements significantly improve the stability and longevity of lyophilized protein drug products, maintaining their efficacy over extended storage periods.

### Lyophilization strategy to minimize product reconstitution time

Long reconstitution times of lyophilized protein drug products pose several problems that can impact both the healthcare providers and patients, as well as the overall perception and utility of the product in a clinical or consumer setting. If not performed correctly, prolonged reconstitution times can impact the stability and efficacy of the protein drug product [80].

Luoma and Lim developed a method to shorten the reconstitution time of a high-concentration IgG1 subclass monoclonal antibody [81]. They explored how reconstitution time is influenced by the vacuum level in the vial’s headspace. They observed a 3-fold decrease in reconstitution time at lower pressures, with times dropping from 75 minutes at higher pressures to about 20 minutes at lower pressures. On the other hand, reducing headspace pressure from 200 Torr to 0.1 Torr cut reconstitution time by at least half in all tests, achieving over 50% reduction in time at pressures below 50 Torr [81].

**Figure 5:**
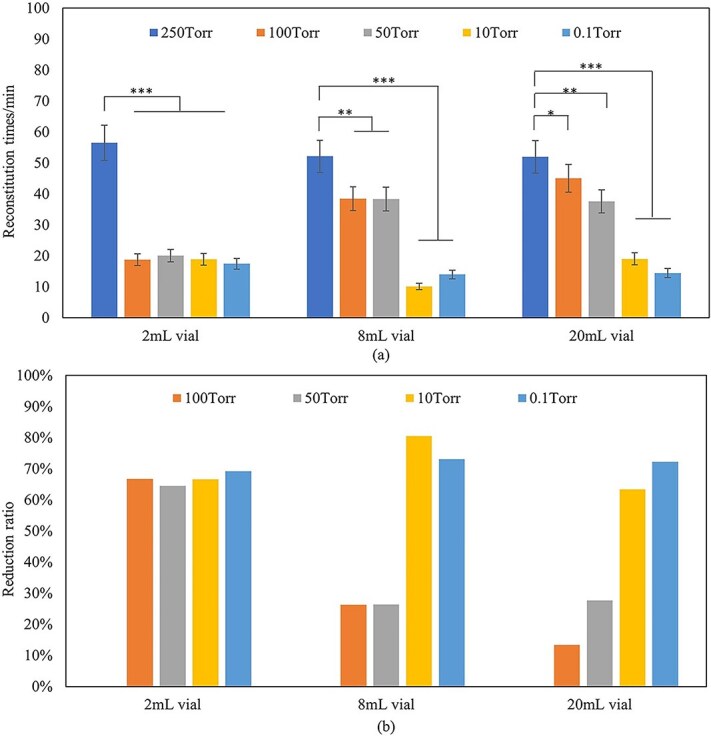
Reconstitution time (a) and reduction ratio (b) of lyophilized formulations (F1, 150 mg/ml protein concentration) with 225 mg protein in 2 ml vial, 450 mg in 8 ml vial, and 900 mg in 20 ml vial at headspace vacuum range from 250 torr to 0.1 torr. N = 10 for each analysis. Error bars represent one standard deviation. Statistically significant differences between the 250 torr, 100 torr, 50 torr, 10 torr, and 0.1 torr cakes are denoted by ^*^(*P* < 0.05), ^*^^*^(*P* < 0.01) and ^*^^*^^*^(*P* < 0.001). Reproduced from [82].

In addition to lowering headspace pressure, Zhang and colleagues suggested adding an annealing step during the lyophilization process to shorten the reconstitution time of lyophilized high-protein concentration drug products [82]. They found that including a −3°C annealing step in the lyophilization cycle could decrease reconstitution time by 38% compared to processes without annealing. Their research also explored how reconstitution time correlates with headspace pressure, revealing those vials with lower headspace pressure reconstitutes faster than those with higher pressure. Specifically, they noted a reduction in reconstitution time of over 60% at headspace pressures below 10 Torr compared to 250 Torr (Fig. 5).

Kulkarni and colleagues reported that crystalline mannitol can significantly decrease the reconstitution time for high concentration protein formulations. It improves the wettability of the cake solids, aids the penetration of reconstitution liquid into the cake’s interior, and makes cakes easier to disintegrate [83].

In summary, effectively managing headspace pressures, incorporating an annealing step and utilizing crystalline mannitol are important strategies for reducing reconstitution time. The annealing step facilitates the formation of larger ice crystals resulting in a lyophilized product with larger pores, making it easier for ice sublimation and reconstitution.

## Summary and future trend

In this review, the scientific foundations, and practical considerations for developing lyophilized protein drug products were illustrated including protein formulation, container/closure and process design for lyophilized drug product. The intent of this review was to consolidate these principles into a set of guidelines for rational design of the lyophilized protein drug product.

To develop an optimal lyophilized protein drug product, it is important for a scientist in pharmaceutical industry to comprehend the goals and requirement of the drug product intended from different stages of clinical use to commercialization. The practical considerations were discussed across both the early and late stages of development, underscoring the necessity of a strategic approach from initial development through to commercialization. Method transfer and lyophilization process scale up are not discussed in this review. The practical advice was given for protein formulation and lyophilization process optimization that are essential for enhancing protein stability and the efficiency of the lyophilization process. In addition, the paper examines the critical elements in selecting primary containers and closures for lyophilized drug products, focusing on vials, stoppers, and dual chamber systems.

New lyophilization technologies are promising to achieve improved drug stability for room temperature storage [84], controlled nucleation technology to improve protein drug product morphology, stability, and reconstitution properties. Advances in lyophilization processes to manufacture a lyophilized protein drug product have targeted moderate moisture content for stability and process efficiency by omitting the secondary drying step [85]. The dual chamber systems offer the potential for at home administration of liable proteins that require the development of high concentration lyophilized drug products.

## Data Availability

No new data were generated or analyzed in support of this review.
